# Elevated Ratio of Urinary Metabolites of Thromboxane and Prostacyclin Is Associated with Adverse Cardiovascular Events in ADAPT

**DOI:** 10.1371/journal.pone.0009340

**Published:** 2010-02-19

**Authors:** Thomas J. Montine, Joshua A. Sonnen, Ginger Milne, Laura D. Baker, John C. S. Breitner

**Affiliations:** 1 Department of Pathology, University of Washington, Seattle, Washington, United States of America; 2 Puget Sound Veterans Affairs Health Care System, Geriatric Research Education and Clinical Center (GRECC), Seattle, Washington, United States of America; 3 Vanderbilt University Medical Center, Nashville, Tennessee, United States of America; Case Western Reserve University, United States of America

## Abstract

Results from prevention trials, including the Alzheimer's Disease Anti-inflammatory Prevention Trial (ADAPT), have fueled discussion about the cardiovascular (CV) risks associated with non-steroidal anti-inflammatory drugs (NSAIDs). We tested the hypotheses that (i) adverse CV events reported among ADAPT participants (aged 70 years and older) are associated with increased ratio of urine 11-dehydrothromboxane B_2_ (Tx-M) to 2′3-donor–6-keto-PGF1 (PGI-M) attributable to NSAID treatments; (ii) coincident use of aspirin (ASA) would attenuate NSAID-induced changes in Tx-M/PGI-M ratio; and (iii) use of NSAIDs and/or ASA would not alter urine or plasma concentrations of F_2_-isoprostanes (IsoPs), *in vivo* biomarkers of free radical damage. We quantified urine Tx-M and PGI-M, and urine and plasma F_2_-IsoPs from 315 ADAPT participants using stable isotope dilution assays with gas chromatography/mass spectrometry, and analyzed these data by randomized drug assignment and self-report compliance as well as ASA use. Adverse CV events were significantly associated with higher urine Tx-M/PGI-M ratio, which seemed to derive mainly from lowered PGI-M. Participants taking ASA alone had reduced urine Tx-M/PGI-M compared to no ASA or NSAID; however, participants taking NSAIDs plus ASA did not have reduced urine Tx-M/PGI-M ratio compared to NSAIDs alone. Neither NSAID nor ASA use altered plasma or urine F_2_-IsoPs. These data suggest a possible mechanism for the increased risk of CV events reported in ADAPT participants assigned to NSAIDs, and suggest that the changes in the Tx-M/PGI-M ratio was not substantively mitigated by coincident use of ASA in individuals 70 years or older.

## Introduction

Prostaglandin (PG) H synthase- or cyclooxygenase (COX) 2 inhibitors, also called coxibs, were developed as an alternative for treatment of arthritis in patients who could not tolerate traditional (t) non-steroidal anti-inflammatory drugs (NSAIDs). No coxib-induced increase in risk of adverse cardiovascular (CV) events had been observed in the relatively short trials of these drugs for treatment of arthritis (reviewed in [1], [2]). Increased rates of adverse CV events began to emerge instead from longer trials of coxibs for the prevention or treatment of other diseases [3]–[5]. In December 2004, increased risk of adverse CV events in groups assigned to celecoxib in the Adenoma Prevention with Celecoxib study resulted in the termination of celecoxib administration in this trial [5]. Upon instruction from the Food and Drug Administration, coxib treatments were halted days later in five other ongoing trials with a coxib treatment arm; one of these was the Alzheimer's Disease Anti-inflammatory Prevention Trial (ADAPT).

It now appears that exposure to coxibs, perhaps even for even as little as two weeks [6], provokes a small but significantly increased risk for adverse CV events that is related in part to the presence of baseline CV risk factors [1], [2]. Although this coxib effect is probably multifactorial, abundant data from experimental models suggest that it derives at in part from suppressed production of prostacyclin (PGI_2_), especially relative to that of thromboxane (Tx) A_2_. Production of TxA_2_ is closely linked to COX-1 activity while production of PGI_2_ is more closely linked to COX-2 activity, with aspirin preferentially inhibiting COX-1 activity and t-NSAIDs inhibiting both COX isozymes without bioactivation [7]–[10].

The degree of CV risk from t-NSAIDs is complicated by several considerations including variable COX isoform inhibition, inter-individual variability in response to drug, interaction with other risk factors for CV disease, and perhaps interaction with aspirin (ASA) used for cardioprotection [2]. Indeed, others have proposed that there are different classes of t-NSAIDs with respect to CV risks, with one class reserved for naproxen because it may provide cardioprotection in some individuals, but also may compete with similar effects of ASA [2]. Naproxen was the t-NSAID used along with celecoxib in ADAPT.

Several of the proposed mechanisms for Alzheimer's disease (AD) pathogenesis can be suppressed by t-NSAIDs or by coxibs in experimental models, and abundant epidemiologic data suggest that the occurrence of AD is reduced among regular users of t-NSAIDs [11] (we know of no such data for coxibs). As a result, several randomized controlled trials have now investigated the effects of NSAIDs on AD pathogenesis [12]. While none of these trials demonstrated benefit for patients with the mild (prodromal) or dementia stages of AD, ADAPT was designed to determine whether naproxen or celecoxib might *prevent* subsequent AD dementia in older individuals with normal cognition at enrollment. Similar to results in the trials described above, however, a small but significant increased risk of adverse CV events also was observed in ADAPT. Somewhat surprisingly, this small risk appeared more evident in participants assigned to naproxen than to celecoxib [13].

When considering the increased CV risk observed in ADAPT participants, it is important to remember that ADAPT differed from the other NSAID trials in that individuals were older (greater than 70 years), and enriched for risk of AD but not for rheumatologic, neoplastic, or CV diseases. Both attributes of ADAPT participants might have influenced baseline CV risk and thereby the risk for adverse CV events from NSAID use. Moreover, unlike some of the previous trials, ADAPT permitted use of cardioprotective ASA, which about half of the relatively more elderly ADAPT cohort used. Here we describe investigation of biomarkers for proposed mechanisms of NSAID-induced adverse CV events in ADAPT participants.

## Methods

ADAPT was a multisite randomized, placebo-controlled, parallel chemoprevention trial conducted in individuals who were 70 years or older and had a family history of AD [13]. Study treatments in ADAPT were celecoxib at a full anti-arthritic dose of 200 mg taken twice daily, and naproxen sodium at the dosage authorized for over-the-counter use: 220 mg taken twice daily. ADAPT participants were permitted voluntary concomitant use of cardioprotective ASA not to exceed 1000 mg/week. We estimated systemic biosynthesis of thromboxane (Tx) A_2_, which derives predominantly from the catalytic action of COX-1, and prostacyclin (PGI_2_), which derives predominantly from the catalytic action of COX-2, by quantifying their respective major urinary metabolites, 11-dehydrothromboxane B_2_ (Tx-M) and 2′3-donor–6-keto-PGF1 (PGI-M) [7]–[9]. We also quantified urine and plasma F_2_-isoprostanes (IsoPs) since these *in vivo* biomarkers of oxidative injury have been proposed by some to derive in part from the action of COX isozymes [14], [15].

Enrollment commenced in March 2001 and administration of ADAPT treatments was suspended on 17 December 2004. We analyzed specimens from all 330 ADAPT participants who had donated fluids before the suspension date. This number included 38 donors who had experienced adverse CV events (see below). We dichotomized the 330 participants according to the self-reported regularity with which they took their assigned treatments. “Compliant” individuals reported that they took their treatments “always or almost always”, “most of the time”, or “about half of the time.” “Non-compliant individuals” reported adherence to their assigned treatment regimen “less than half the time”, “infrequently”, or “never”. Fourteen individuals did not provide data on their treatment compliance, and one sample from the “compliant” group was lost. Therefore, we determined the concentrations of the three biomarkers in samples from the remaining 315 participants.

Tx-M, PGI-M, and F_2_-IsoPs were quantified using stable isotope dilution assays with gas chromatography/mass spectrometry, as previously described [9], [16]. Results below the limit of detection for any analyte were assigned a value of zero for that analyte. Occasional spectra were not resolvable and were treated as missing. This resulted in 23 missing Tx-M samples and 20 missing PGI-M samples. Urine concentrations were expressed in ng/mg of creatinine. Statistical analyses were performed initially using GraphPad Prism version 4.03 (GraphPad Software, San Diego, CA) with α = 0.05, and confirmed in Stata, version 10 (STATA Corporation, College Station, TX).

## Results

### Participant Characteristics

The compliant (n = 190) and non-compliant (n = 125) groups did not differ with respect to age (mean ± SD: 76±4 vs. 77±4 years; P>0.05), gender (89 women and 101 men in the compliant group, vs. 70 and 55 in non-compliant participants; χ^2^ test; P>0.05), education (mean ± SD: 15±3 years for both), or presence of the *APOE* ε4 allele (18% vs. 22%; P>0.05).

### Associations of NSAID and ASA Use with COX-Derived Metabolites

Data were considered in separate two-way ANOVAs for urinary Tx-M or PGI-M concentrations in compliant and non-compliant participants, some of whom also voluntarily took ASA (**Table 1**). Average age ± SD and % female for ASA users were 77±4 years and 55% female, and for ASA non-users were 76±4 and 56% female. Treatment group assignment was a statistically significant predictor of urine Tx-M and PGI-M concentrations in the compliant group (P<0.0001 for each group). Urine Tx-M and, to a lesser degree, urine PGI-M concentrations also were reduced significantly with ASA use in the compliant group (P<0.0001 and P<0.05). Among compliant subjects, there was a significant statistical interaction between treatment assignment and ASA use for urinary concentration of Tx-M, but not of PGI-M. This interaction reflected a significant additional suppressive effect of ASA use on Tx-M levels in subjects compliant to placebo or celecoxib (P for either<0.0001), but not naproxen. As expected, the non-compliant group showed no significant effect of treatment assignment on either urine Tx-M or PGI-M concentrations (P>0.05 for both). However, non-compliant participants who were ASA users did show significantly reduced concentrations of Tx-M but not PGI-M (P<0.05). There was no evidence of a statistical interaction between group assignment and ASA use among non-compliant participants. In summary, we observed the expected changes in urine Tx-M and PGI-M concentrations by treatment assignment and by ASA use among compliant ADAPT participants, and by ASA use among non-compliant donors.

**Table 1:** Urine Tx-M and PGI-M concentrations in ADAT participants categorized by treatment adherence, group assignment, and aspirin use.

Urine eicosanoid	Adherence	Assignment	No Aspirin (n)	Aspirin (n)
Tx-M (ng/mg Cr)	Compliant^+^	Placebo	0.45+0.04 (27)	0.22+0.02 (52)^#,^ *
		Naproxen	0.23+0.05 (29)	0.17+0.02 (27)^#^
		Celecoxib	0.40+0.04 (16)	0.16+0.02 (26)^#,^ *
		**Subtotal**	**0.35+0.03 (72)**	**0.19+0.01 (105)^++^**
	Non-compliant^∧^	Placebo	0.43+0.07 (20)	0.29+0.09 (21)^#^
		Naproxen	0.36+0.07 (13)	0.20+0.03 (26)^#^
		Celecoxib	0.35+0.06 (10)	0.21+0.03 (25)^#^
		**Subtotal**	**0.39+0.04**	**0.23+0.03^++^**
PGI-M (ng/mg Cr)	Compliant^+^	Placebo	0.23+0.02 (29)	0.16+0.02 (50)^#^
		Naproxen	0.11+0.02 (26)	0.10+0.03 (27)^#^
		Celecoxib	0.12+0.02 (18)	0.06+0.01 (29)^#^
		**Subtotal**	**0.16+0.01 (73)**	0.12+0.01 (106)**
	Non-compliant^∧^	Placebo	0.18+0.03 (19)	0.15+0.04 (21)
		Naproxen	0.15+.02 (14)	0.13+0.02 (26)
		Celecoxib	0.13+0.02 (9)	0.12+0.02 (27)
		**Subtotal**	**0.16+0.01 (42)**	0.13+0.01 (74)**

Two-way ANOVAs were performed for each eicosanoid in compliant and non-compliant subjects for the three treatment groups. Group assignment was significantly (^+^P<0.0001) related to urine Tx-M and PGI-M in the compliant group, but not for the non-compliant group (^∧^P>0.05). ASA use was associated with reduced urine Tx-M concentrations (^#^P<0.05) in the compliant and non-compliant groups, and reduced urine PGI-M in only the compliant group (^#^P<0.05). A significant interaction between group assignment and ASA use (P<0.05) was detected only for urine Tx-M concentrations in the compliant group. Bonferroni-corrected post-hoc comparisons evaluated the effects of ASA use in each of the twelve groups; only urine Tx-M in compliant Placebo and compliant celecoxib participants was significant (*P<0.0001) while all others had P>0.05. In addition, values for ASA compliant and noncompliant individuals were calculated regardless of treatment group assignment, presented in rows labeled subtotal, and compared using t-tests (^++^P<0.0001, **P<0.05).

### Relation of Prostaglandin Metabolites to Serious Cardiovascular Adverse Events

We contrasted urine Tx-M, urine PGI-M, and the ratio of these two analytes in the 38 individuals with a composite cardiovascular endpoint including cardiovascular death, myocardial infarction, or stroke (CV+) vs. the other 277 participants without such events (CV−). Average age ± SD and % female for the CV+ group were 77±3 years and 42% female, and for the CV− group were 76±4 years and 51% female. Non-parametric Mann-Whitney t-tests showed that urinary PGI-M was significantly lower in the CV+ group (0.10±0.01 ng/mg Cr) than in the CV− group (0.15±0.01 ng/mg Cr; P = 0.01). Urine Tx-M levels also tended to be higher in CV+ individuals, but these values did not differ significantly from those of CV− subjects. As predicted, the ratio of urine Tx-M/PGI-M was significantly higher in the CV+ group (3.45±0.43 ng/mg Cr) vs. CV− group (3.08±0.29 ng/mg Cr; P<0.05). Although the number of adverse events in the cohort was relatively small, these data suggest that higher urine Tx-M/PGI-M ratio was associated with serious adverse CV events in ADAPT participants.

**Figure 1:**
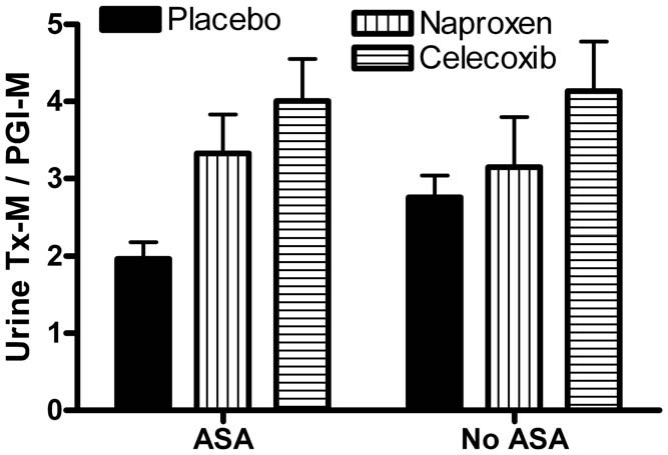
Urine Tx-M/PGI-M ratio in compliant ADAPT participants who self-reported ASA use was analyzed by two-way ANOVA; treatment group assignment (P<0.01), but not ASA use significantly affected this ratio. Bonferroni-corrected post-hoc t-tests showed that among treatment groups, only Placebo was significantly different (P<0.05) between ASA and No ASA users.

We sought also to determine whether co-incident use of ASA would ameliorate NSAID-induced shifts in the urine Tx-M/PGI-M ratio (**Figure 1**). Here we restricted our analysis to compliant participants. Treatment group assignment (P = 0.001), but not ASA use (P>0.05) significantly affected urine Tx-M/PGI-M ratio. Bonferroni-corrected post-hoc paired comparisons showed that placebo had significantly lower ratios than naproxen- or celecoxib-assigned groups who were also voluntary ASA users (P<0.05 and <0.001, respectively). Although a similar trend was observed among participants who did not use ASA, these differences were not statistically significant. When comparing the effects of ASA use within treatment groups, only placebo-assigned ASA users had a significantly (P<0.05) lower ratio than did participants who did not report use of ASA. Although not originally specified as part of the trial design and relying on self-reported ASA use, these results suggest that co-incident use of ASA did not offset celecoxib-induced changes in urine Tx-M/PGI-M ratio in ADPAT participants.

### F_2_-IsoPs Concentrations

The concentrations of urine and plasma F_2_-IsoP were significantly correlated (P<0.01), but neither showed a significant relationship to treatment assignment or ASA use in compliant or non-compliant participants. Among compliant participants, plasma F_2_-IsoP concentrations were 52±9 pg/ml (average ± SEM) for the 27 placebo-assigned subjects who did not use ASA vs. 60±5 pg/ml for the 47 who did use ASA. In the urine, F_2_-IsoP concentrations were 2.2±0.3 ng/mg Cr for 29 compliant placebo-assigned subjects who did not use ASA vs. 2.0±0.1 ng/mg Cr for the 52 who did use ASA. There was no evidence for an interaction between treatment group assignment and ASA use for F_2_-IsoPs in either plasma or urine. In summary, neither NSAIDs nor ASA had a significant effect on plasma or urine concentrations of this quantitative *in vivo* measure of free radical damage in ADAPT participants.

## Discussion

We undertook analyses of urine and plasma samples from ADAPT participants to determine whether naproxen or celecoxib treatment in individuals 70 years or older produced changes in urine Tx-M and PGI-M levels that were similar to findings reported previously in younger individuals. We also sought to test three hypotheses: (i) that CV adverse events in ADAPT participants were associated with an increase in the Tx-M/PGI-M ratio, (ii) that use of ASA might ameliorate NSAID-induced changes in urine Tx-M/PGI-M ratio, and (iii) that use of NSAIDs and/or ASA would alter urine or plasma F_2_-IsoP concentrations.

Production of TxA_2_ is closely linked to COX-1 activity while production of PGI_2_ is more closely linked to COX-2 activity [7]–[10]. Our results showed relative suppression of the major urinary metabolites, Tx-M or PGI-M, that varied with selective inhibition of COX isoforms by these drugs. In addition, serious adverse CV events in ADAPT participants were significantly associated with a higher urine Tx-M/PGI-M ratio, which in turn seemed to derive mainly from lowered PGI-M. These were the expected results based on previous studies and exclude the possibility of unexpected pharmacologic activity in this older cohort. However, increased CV risk in ADAPT was observed more in the t-NSAID (naproxen) than in the coxib group. While it is true that both of these ADAPT treatment groups had higher urine Tx-M/PGI-M ratio compared to placebo, the highest values were observed with coxib users. As is now clear from several studies, adverse CV events from NSAID use are multifactorial and dependent in part on baseline CV risk factors that may not have been adequately balanced by the randomization process in ADAPT. Unfortunately, because CV events were not originally intended as an outcome measure in ADAPT, we do not have good data on CV risks in participants at baseline. It is therefore possible that some combination of drug-induced changes and a lack of balance in baseline CV risk factors accounts for small increased CV risks that appeared among naproxen users in ADAPT.

It has been suggested that co-incident treatment with ASA might protect against unfavorable NSAID-induced shifts in the production of TxA_2_ and PGI_2_. ADAPT was not designed to test effects of ASA (use was not randomized), so our data are only suggestive. They do, however, indicate that participants taking NSAIDs and ASA did *not* have a reduced urine Tx-M/PGI-M ratio. To the extent that the cardioprotection from ASA use is reflected in the Tx-M/PGI-M ratio, therefore, our data suggest that the cardioprotective effects of ASA are modest in individuals using NSAIDs according to the ADAPT protocol.

F_2_-IsoPs are widely used, validated quantitative *in vivo* measures of free radical damage [17]. Some have proposed that F_2_-IsoPs also can derive from COX activity, thereby complicating the interpretation of changes in concentrations of these biomarkers [14], [15]. However, unlike the two COX-derived metabolites discussed above, plasma and urine F_2_-IsoP concentrations were not significantly changed with use of celecoxib, naproxen, or ASA in ADAPT participants. We conclude that, similar to findings in animal models [17], COX-induced production of F_2_-IsoPs occurs at undetectable levels in older humans.

Our results showed that, similar to results reported from younger cohorts, ADAPT participants who were compliant with their assigned treatment regimen showed expected changes in urine Tx-M and PGI-M, but not in plasma or urine F_2_-IsoPs. Moreover, as expected, adverse CV events were associated with an increased Tx-M/PGI-M ratio. Importantly, however, our results also suggested that these effects were not materially modified with self-reported voluntary use of ASA. Our data thus provide a partial mechanistic explanation for the increase risk for CV events reported in ADAPT, but they also suggest that this risk from NSAID use might not be substantively mitigated by co-administration of ASA, at least in those over 70 years of age.
